# Inducible cysteine synthesis by CysK2 provides copper resistance in *Mycobacterium tuberculosis*

**DOI:** 10.1128/mbio.00888-26

**Published:** 2026-07-29

**Authors:** Daisy X. Ji, Nicholas R. Silvaggi, Anna Benko, K. Heran Darwin

**Affiliations:** 1 Department of Microbiology, NYU Grossman School of Medicine 12296 , New York, New York, USA; 2 Department of Chemistry and Biochemistry, University of Wisconsin-Milwaukee 14751, Milwaukee, Wisconsin, USA; Washington University in St. Louis, St. Louis, Missouri, USA

**Keywords:** *Mycobacterium tuberculosis*, copper sensing, cysteine biosynthesis

## Abstract

Copper is an essential nutrient that at high levels is toxic to life. Mammalian hosts can accumulate copper in phagolysosomes to restrict a variety of pathogens, including *Mycobacterium tuberculosis*. However, pathogens have evolved various mechanisms to counter copper stress. While several pathways that mitigate copper-derived toxicity have been described in *Mycobacterium tuberculosis*, the *Mycobacterium tuberculosis* lab strain H37Rv is significantly more sensitive to copper than other lab strains, including CDC1551 and Erdman. Here, we determine a cause of this sensitivity in H37Rv is due to a single amino acid substitution in CysK2 (Rv0848), which is encoded in the copper-inducible RicR regulon required for robust copper resistance and virulence. H37Rv CysK2 was previously reported as a S-sulfocysteine synthase. However, we found CysK2 is a cysteine synthase, the activity of which is required for resistance to copper. This study is the first to link *de novo* cysteine biosynthesis with copper resistance in *Mycobacterium tuberculosis*.

**IMPORTANCE:**

Copper is a proposed component of the host immune response against *Mycobacterium tuberculosis*; thus, an understanding of how this pathogen mitigates copper stress may yield new targets for therapeutics. Cysteine synthesis is implicated in copper responses in other bacterial species, but had not been shown in mycobacterial copper resistance. This study demonstrates for the first time that the *Mycobacterium tuberculosis* cysteine synthase CysK2 and, by extension, cysteine biosynthesis, can promote copper resistance.

## INTRODUCTION

*Mycobacterium tuberculosis* (*Mtb*), the etiological agent of tuberculosis (TB), is the leading cause of death by a single infectious agent worldwide (1). The only approved vaccine does not protect against the most common pulmonary infections. Antibiotic treatment for TB is lengthy, and antibiotic resistance is steadily rising (1). To attempt to control *Mtb* growth, a host’s immune system uses a variety of toxic factors, such as nitric oxide (2), reactive oxygen species (3, 4), aldehydes (5, 6), and copper (7, 8). While copper is an essential metal-cofactor in both eukaryotes and prokaryotes (9, 10), at high levels, it can be toxic. A variety of bacterial pathogens, including *Mtb*, encounter copper during infections (reviewed in reference 10). Macrophages infected with *Mtb* or activated with tumor necrosis factor alpha (TNF-α) or interferon-gamma (IFNγ) accumulate copper within bacteria-containing phagolysosomes (11). The mammalian phagolysosomal copper transporter ATP7A is required for copper-dependent control of *Escherichia coli* (12) and *Salmonella enterica* Typhimurium (13). How copper controls bacterial growth is incompletely understood, although several mechanisms have been demonstrated, including directly displacing iron from iron-sulfur clusters (14, 15), inducing protein aggregation (16), and generating oxidative stress through a Fenton-like reaction (17).

Although copper is potentially toxic, it is also essential for most life forms. Consequently, copper homeostasis is critical for survival. Toxicity can be avoided when copper is associated either with other elements, small molecules, or proteins. In the presence of excess copper, bacterial pathogens have evolved varying copper resistance systems. In *Mtb*, there are two copper-responsive regulons: CsoR (copper sensitive operon repressor) (18, 19); and RicR (regulated in copper repressor), which is unique to pathogenic mycobacteria (7, 20, 21). In addition, MctB (Rv1698), a proposed membrane porin (8), and transcriptional signaling through Rip1-PtdaR/S (22) contribute to copper homeostasis in *Mtb. Mtb* strains deficient in *mctB* or *ptdaR*/S or producing a RicR allele that cannot respond to copper are all significantly attenuated in animal models (7, 8, 22).

It has been reported that the commonly studied *Mtb* strain H37Rv is more sensitive to copper than two other common lab strains, Erdman (discussed in reference 22) and CDC1551 (7). This observation suggested that there are differences in H37Rv that contribute to increased sensitivity to copper. In this study, we noted the presence of a single-nucleotide polymorphism (SNP) in H37Rv *cysK2* that results in a glycine substitution of a highly conserved serine at position 93 (S93G), with serine as the likely ancestral residue. Expression of *cysK2_S93_* in H37Rv significantly increased copper resistance. We further showed that CysK2 catalyzes the conversion of *O*-phospho-L-serine to cysteine. Unlike other characterized cysteine synthase genes in *Mtb*, expression of *cysK2* is repressed by RicR and, therefore, copper inducible (20). Taken together, our data support a model whereby copper induces the expression of a cysteine synthetic pathway in *Mtb* to mitigate copper toxicity.

## RESULTS

### *Mtb* H37Rv is more sensitive to copper than Erdman and CDC1551 due to an amino acid substitution in CysK2

**Fig 1:**
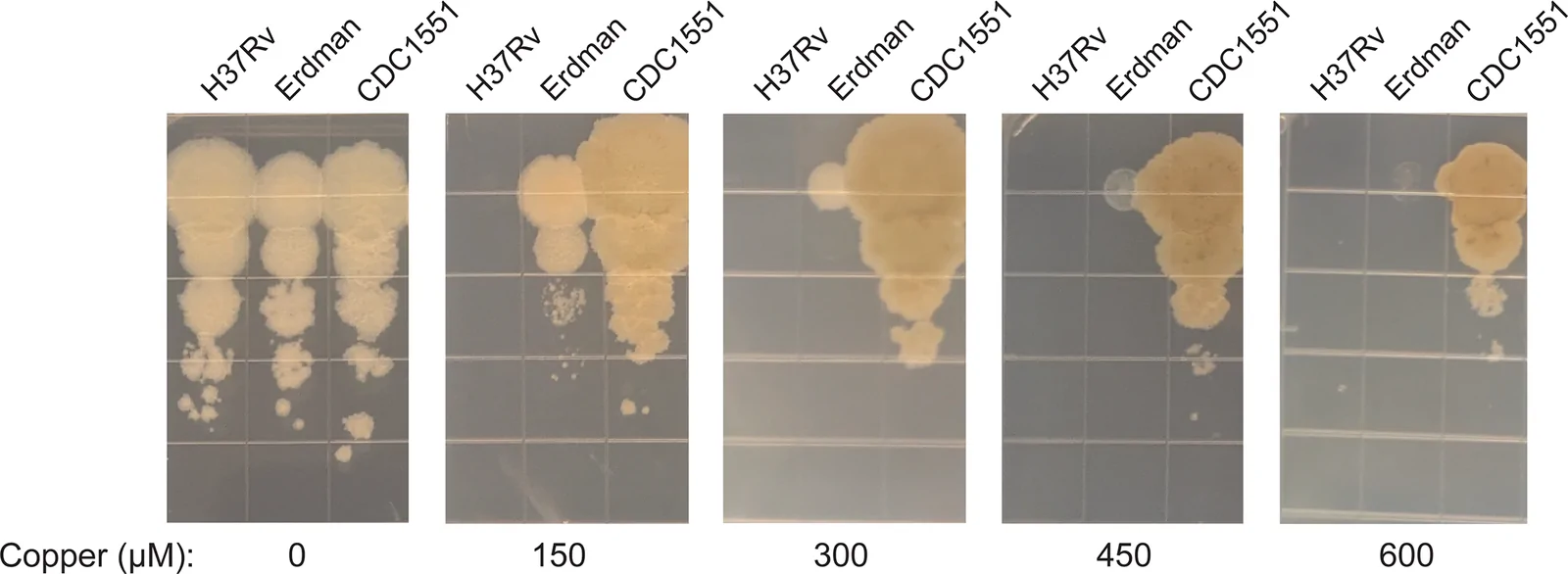
*Mtb* H37Rv is more sensitive to copper than Erdman and CDC1551. Bacteria were spotted onto 7H11 agar plates supplemented with CuSO_4_ at the indicated concentrations. Data are representative of two independent experiments.

*Mtb* H37Rv is more sensitive to copper sulfate (CuSO_4_) than another commonly studied laboratory strain, CDC1551 (7). We confirmed this phenotype and found H37Rv was also significantly more copper-sensitive than another commonly used laboratory strain, Erdman (Fig. 1).

**Fig 2:**
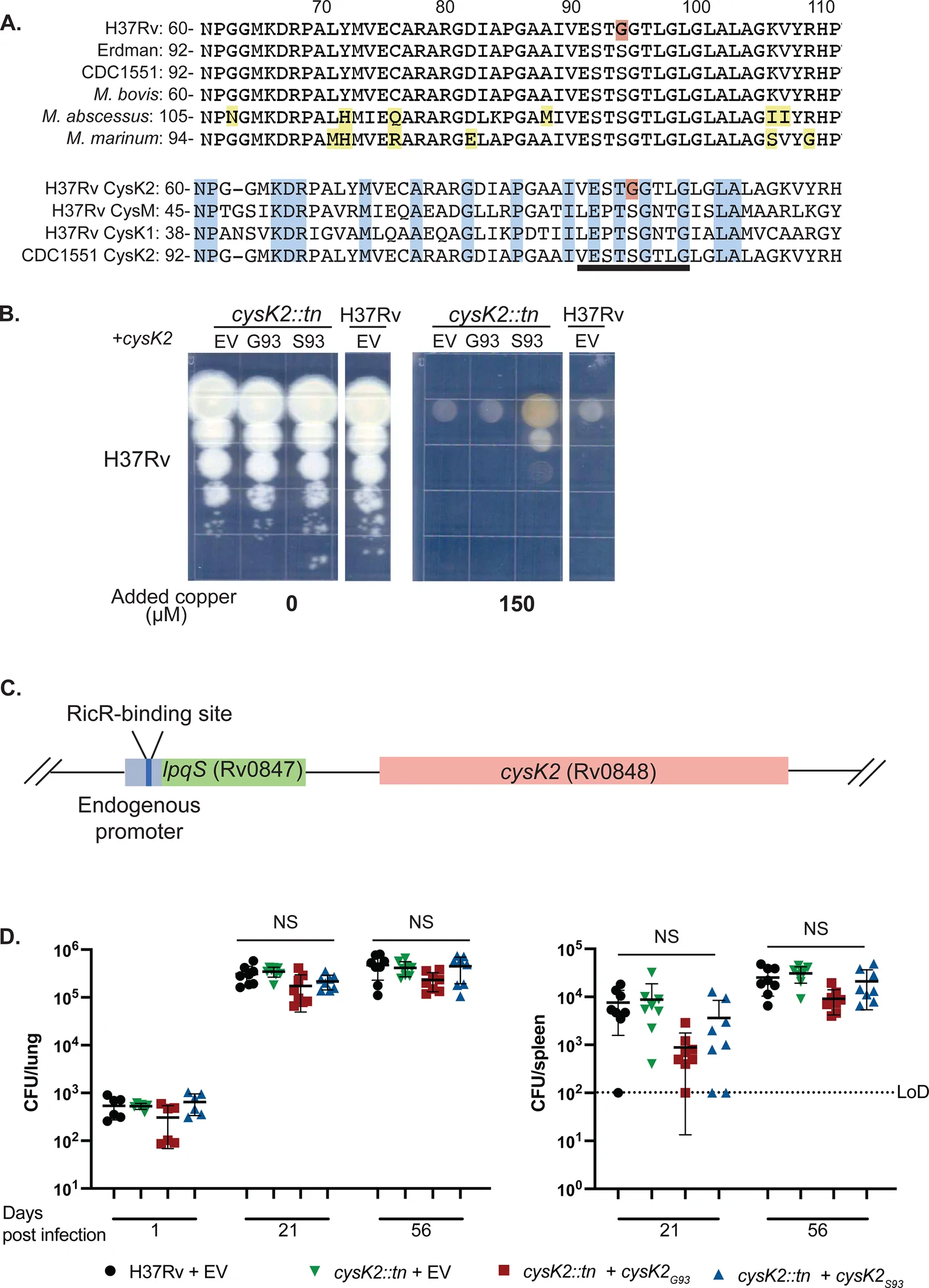
H37Rv carries a S93G substitution in CysK2 that disrupts copper resistance. (**A**) Top: alignment of CysK2 sequences from H37Rv, Erdman, CDC1551, *M. bovis*, *M. abscessus*, and *M. marinum*. S93G substitution in H37Rv CysK2 is highlighted in red. Differences from the consensus sequence are highlighted in yellow. Bottom: alignment of CysK2, CysM, and CysK1 from H37Rv and CysK2 from CDC1551. Regions of identical sequences are marked in blue. A region (23) involved in interaction with the substrate carboxyl group is indicated by the black bar. (**B**) Copper sensitivity assay of parental H37Rv transformed with empty vector pMV306.strep ("EV") and H37Rv *cysK2::tn* transformed with pMV306.strep (“EV”), pMV305.strep-*cysK2_G93_* ("G93"), or pMV306.strep-*cysK2_S93_* ("S93"). Results are representative of three independent experiments. (**C**) Schematic diagram of pMV306.strep-*cysK2* plasmid (see Table 1). Backbone of the pMV306.strep has been omitted for simplicity. (**D**) Aerosol infection of C57BL/6 mice with the same strains as in panel** B**. Data combined from two independent infections. Limit of detection is indicated by a dotted line (“LoD”). Kruskal-Wallis test was used for statistical analysis. Error bars show mean and SEM.

We next compared the sequences of genes previously associated with copper resistance, looking for SNPs that distinguish H37Rv from CDC1551 and Erdman (see Methods). We found the RicR-regulated gene *cysK2* in H37Rv has an adenine-to-guanine substitution resulting in a glycine instead of serine at position 93 when compared to Erdman and CDC1551 (Rv0848; Fig. 2A, top). Alignment with other mycobacteria with an annotated *cysK2* showed that serine is highly conserved at this position (Fig. 2A, top). Alignment with the two other cysteine synthases in *Mtb* (CysM and CysK1) showed that the amino acid at position 93 corresponds to a region that typically coordinates the substrate’s carboxyl group (23), and the equivalent positions in CysM and CysK1 are also serine (Fig. 2A, bottom). Together, these results suggest that G93 in H37Rv CysK2 might result in an attenuation of function.

To determine if the S93G difference in CysK2 contributes to increased copper susceptibility of H37Rv, we compared an H37Rv *cysK2* transposon insertion mutant (*cysK2*::MycoMarT7 or "*cysK2::tn*") complemented with either its native *cysK2_G93_* or with *cysK2_S93_* using a semi-quantitative, agar plate-based assay. *cysK2::tn* strains grew similarly as the parental H37Rv strain on agar plates with trace levels of copper (6 µM) found in 7H11 media (see Table 1 for strains used in this study; Fig. 2B). Both parental and *cysK2::tn* strains were severely growth-restricted on agar supplemented with 150 µM CuSO_4_, and the addition of *cysK2_G93_* to the *cysK2::tn* mutant did not increase copper resistance (Fig. 2B). In contrast, *cysK2::tn* complemented with *cysK2_S93_* under the control of its native promoter showed a substantial increase in copper resistance (Fig. 2B). *cysK2* expression is repressed at a promoter upstream of *lpqS* by RicR (20), and we found successful complementation required inclusion of *lpqS*, possibly due to the presence of sequence elements needed for *cysK2* translation. Therefore, all *cysK2* complementation analyses used in this study included *lpqS* (see Table 1 for plasmids and primers used and Fig. 2C). The shared phenotype of H37Rv, *cysK2::tn*, and *cysK2::tn + cysK2*_G93_ suggests that CysK2_G93_ is possibly defective, and that H37Rv is a natural mutant of *cysK2*. Therefore, CysK2_S93_ is likely the ancestral or wild-type enzyme, and CysK2_G93_ is a divergent, mutant allele specific to H37Rv.

**TABLE 1:** Strains, plasmids, and primers used in this study

Strain, plasmid, or primer	Genotype, phenotype, or sequence	Source or purpose
Strains		
H37Rv	*M. tuberculosis* "wild type"	American Type Culture Collection 25618
CDC1551	*M. tuberculosis* "wild type"	Bishai lab collection
Erdman	*M. tuberculosis* "wild type"	BEI Resources, NIAID, NIH. Strain Erdman K01 (TMC 107), NR-15404
MHD761	H37Rv pMV306.strep	Samanovic et al. (24)
MHD1901	H37Rv Kan^r^ Strep^r^ *cysK2*::ΦMycoMarT7/pMV306.strep-*cysK2_S93_*	This study
MHD1902	H37Rv Kan^r^ Strep^r^ *cysK2*::ΦMycoMarT7/pMV306.strep-*cysK2_G93_*	This study
MHD1905	H37Rv Kan^r^ Strep^r^ *cysK2*::ΦMycoMarT7/pMV306.strep	This study
MHD1965	H37Rv Hyg^r^ Strep^r^ *∆cysK2::hyg*/pMV306.strep	This study
MHD1991	H37Rv Hyg^r^ Strep^r^ *∆cysK2::hyg*/pMV306.strep-*cysK2_S93_*	This study
MHD1990	H37Rv Hyg^r^ Strep^r^ *∆cysK2::hyg*/pMV306.strep-*cysK2_G93_*	This study
MHD1968	Erdman Hyg^r^ Strep^r^ *∆cysK2::hyg*/pMV306.strep	This study
MHD1993	Erdman Hyg^r^ Strep^r^ *∆cysK2::hyg*/pMV306.strep-*cysK2_S93_*	This study
MHD1992	Erdman Hyg^r^ Strep^r^ *∆cysK2::hyg*/pMV306.strep-*cysK2_G93_*	This study
MHD1971	CDC1551 Hyg^r^ Strep^r^ *∆cysK2::hyg*/pMV306.strep	This study
MHD1995	CDC1551 Hyg^r^ Strep^r^ *∆cysK2::hyg*/pMV306.strep-*cysK2_S93_*	This study
MHD1994	CDC1551 Hyg^r^ Strep^r^ *∆cysK2::hyg*/pMV306.strep-*cysK2_G93_*	This study
DH5α	*E. coli* cloning strain; θ80Δ*lac*ZM15 Δ(*lacZYA-argF*)U169 *deoR recA1 endA1hsdR17* (r_k_-m_k_+) *phoA supE44* λ- *thi-1 gyrA96 relA1*	Gibco, BRL
BL21 Star (DE3)	*E. coli* expression strain; F^–^*ompT hsdSB* (rB–, mB–) *gal dcm rne131*λ(DE3)	Thermo Fisher/Invitrogen
Plasmids		
pMV306.strep	Strep^r^, mycobacterial plasmid that integrates at *attB* site on the *Mtb* chromosome	Gift from John McKinney
pMV306.strep-*cysK2_S93_*	Strep^r^, for complementation with *cysK2_S93_*; includes RicR-binding site and *lpqS* at 5′ end (see text)	This study
pMV306.strep-*cysK2_G93_*	Strep^r^, for complementation with *cysK2_G93_*; includes RicR-binding site and *lpqS* at 5′ end (see text)	This study
pYUB854	Hyg^r^, allelic-exchange vector	Bardarov et al. (25)
pYUB854-Δ*cysK2*	Hyg^r^, *M. tuberculosis ∆cysK2::hyg* (deletion-disruption plasmid)	This study
pQE-30	Amp^r^, for production of N-terminally 6×His-tagged protein	Qiagen
pQE-30-*cysK2_S93_*	Amp^r^, for production of His_6_-TEV-site-CysK2_S93_ in *E. coli*	
pQE-30-*cysK2_G93_*	Amp^r^, for production of His_6_-TEV-site-CysK2_G93_ in *E. coli*	
pE-SUMOpro(kan)	Kan^r^, for production of N-terminally SUMO epitope-tagged protein	LifeSensors, Inc.
pSUMO-*cysK2_S93_*	Kan^r^, for production of SUMO-CysK2_S93_ in *E. coli*	This study
pSUMO-*cysK2_G93_*	Kan^r^, for production of SUMO-CysK2_G93_ in *E. coli*	This study
Primers		
CysK2_F1	GCTAGCACATCGGGCAGTACGCAAC	For cloning *cysK2* (NheI)
CysK2_R1	TTACGACACCACCTGGGTTG	For cloning *cysK2* (blunt)
CysK2_seq1	CCCAACAAGCATCACCTCCAAC	For sequencing *cysK2*
CysK2_seq2	GGATCGATGTCCTGGTGTGCTC	For sequencing *cysK2*
CysK2_KO_F1	CTTAAGTGGATTTGGTCTCGCTCTTCGT	For making *cysK2* deletion mutant (AflII)
CysK2_KO_R1	TCTAGACCGGCCAGGAGTGACTTGATAT	For making *cysK2* deletion mutant (XbaI)
CysK2_KO_F2	CCATGGTACTGCAACGAACACGAACTGC	For making *cysK2* deletion mutant (NcoI)
CysK2_KO_R2	AAGCTTAGATCTGGAACGACAGCGCATA	For making *cysK2* deletion mutant (HindIII)
CysK2_pQE_TEV_F1	GGATCCgagaacctgtacttccaaggtGTGAGGTCGCGGCAGAC	For cloning *cysK2* with an N′ TEV-cleavage site into pQE30 (BamHI)
CysK2_pQE_TEV_R1	AAGCTTTTACGACACCACCTGGGTTG	For cloning *cysK2* with an N′ TEV-cleavage site into pQE30 (HindIII)
CysK2_pSUf	GGTCTCAAGGTGTGAGGTCGCGGCAGAC	For subcloning *cysK2* (BsaI)
CysK2_pSUr	GCTCTAGATCATTACGACACCACCTGGGTTG	For subcloning *cysK2* (XbaI)

We infected C57BL/6 mice by the aerosol route with a medium infection dose (~400 colony forming units, CFU, per mouse) of H37Rv or the *cysK2::tn* mutant transformed with empty vector (pMV306.strep), or *cysK2::tn* pMV-*cysK2_G93_* or *cysK2_S93_* (see Table 1 and Materials and Methods for details). All strains resulted in similar bacterial burdens, suggesting *cysK2* does not play a significant role in H37Rv virulence under the conditions tested (Fig. 2D).

### Deletion of *cysK2* in *Mtb* strains Erdman and CDC1551 increases sensitivity to copper

**Fig 3:**
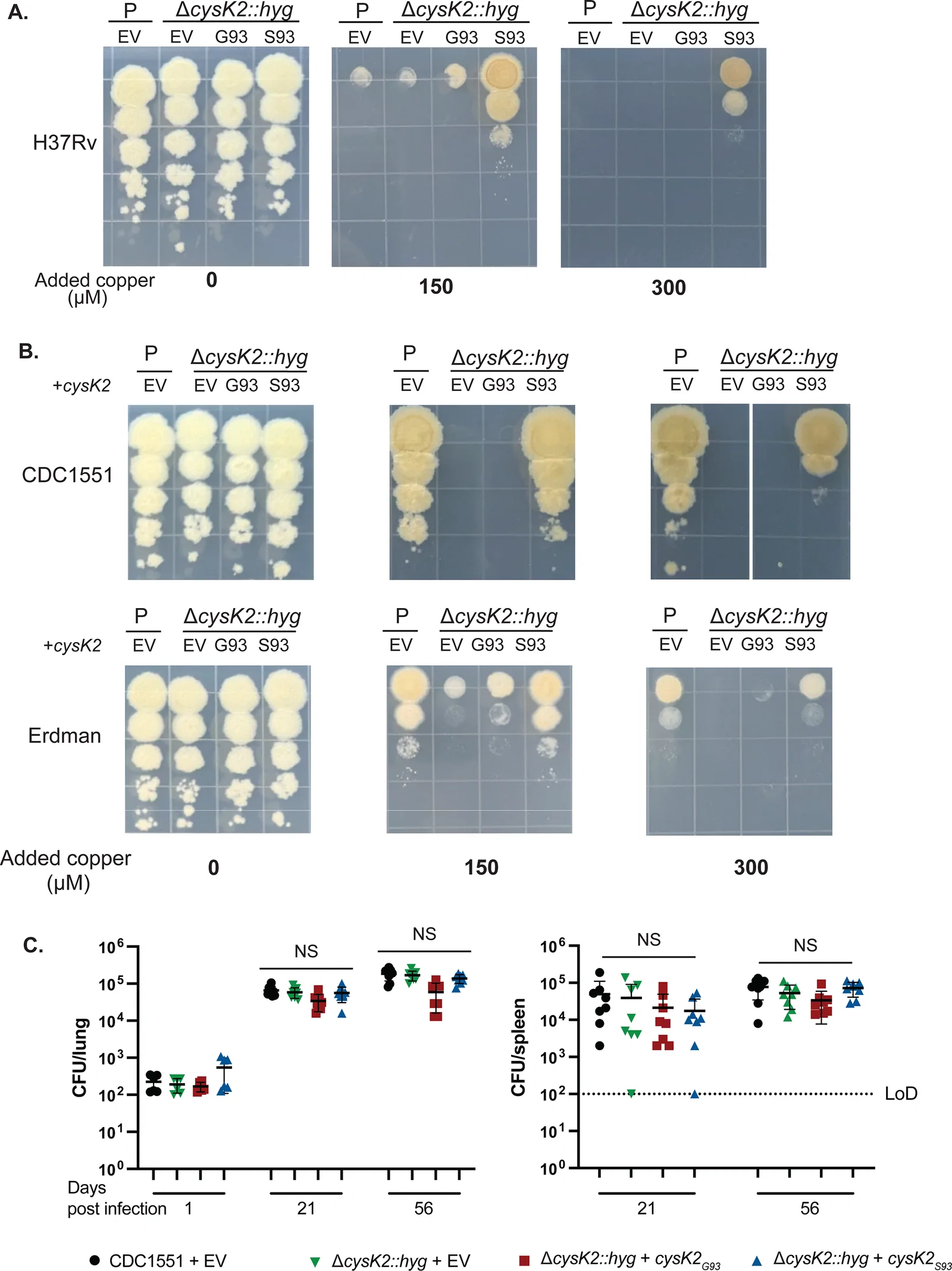
Deletion of *cysK2* in Erdman and CDC1551 increases sensitivity to copper. (**A**) Copper sensitivity assay with parental H37Rv (“P”) transformed with empty vector pMV306.strep ("EV"), or H37RvΔ*cysK2* transformed with EV, pMV306.strep-*cysK2_G93_* ("G93") or pMV306.strep-*cysK2_S93_* ("S93"). Results representative of two independent experiments. (**B**) Copper sensitivity assay with CDC1551 and Erdman and isogenic Δ*cysK2* strains. For both backgrounds, parental strains (“P”) transformed with empty vector pMV306.strep (EV) and Δ*cysK2* strains transformed with EV, pMV306.strep-*cysK2_G93_* ("G93"), or pMV306.strep-*cysK2_S93_* ("S93"). Results are representative of two independent experiments. (**C**) Aerosol infection of mice with the CDC1551 strains used in panel **B**. Data are combined from two independent experiments. Limit of detection is indicated by a dotted line (“LoD”). Kruskal-Wallis test was used for statistical analysis. Error bars show mean and SEM.

To further examine the role of CysK2_S93_, we deleted and replaced *cysK2* from *Mtb* Erdman and CDC1551 strains by allelic exchange. As a control, we used the same method to delete and replace *cysK2* from H37Rv. In all three strains, we replaced the majority of the *cysK2* open reading frame with a hygromycin resistance cassette (Δ*cysK2::hyg* or "Δ*cysK2*") using pYUB854 (20, 25). As observed with the transposon insertion mutant, H37RvΔ*cysK2* grew comparably to parental H37Rv on copper-supplemented agar, and complementation of this mutation with *cysK2_S93_* increased copper resistance of H37Rv (Fig. 3A). In contrast, deletion of *cysK2* from CDC1551 or Erdman resulted in a dramatic reduction in copper resistance, a phenotype that was complemented by *cysK2_S93_* (Fig. 3B). Complementation with *cysK2_G93_* led to a slight increase in copper resistance in H37Rv (Fig. 3A) and Erdman (Fig. 3B, bottom), but not in CDC1551, on plates with 150 μM added CuSO_4_.

We infected mice by aerosol with ~200 CFU/mouse of CDC1551Δ*cysK2* transformed with either *cysK2_S93_*, *cysK2_G93_* , or empty vector. Similar to what we observed with H37Rv *cysK2::tn*, none of these strains showed differences in bacterial burden in mice from the parental CDC1551 strain under the conditions tested (Fig. 3C).

### CysK2 is a cysteine synthase

A previous study showed that purified CysK2_G93_ produces S-sulfocysteine from O-phospho-L-serine (OPS) and thiosulfate *in vitro* (23). While the authors noted that CysK2 from other strains of *Mtb* had serine at position 93, they did not test the effect of this allelic difference on enzyme activity and the resulting product. To investigate how this mutation affects enzyme activity, we cloned *cysK2_G93_* and *cysK2_S93_* with SUMO tags for overproduction in *E. coli* (see Table 1 for plasmids and strains). Both proteins were produced at similar levels in *E. coli*, with each yielding approximately 10 mg/L^−1^ of culture, suggesting that the S93G substitution does not affect protein stability.

**Fig 4:**
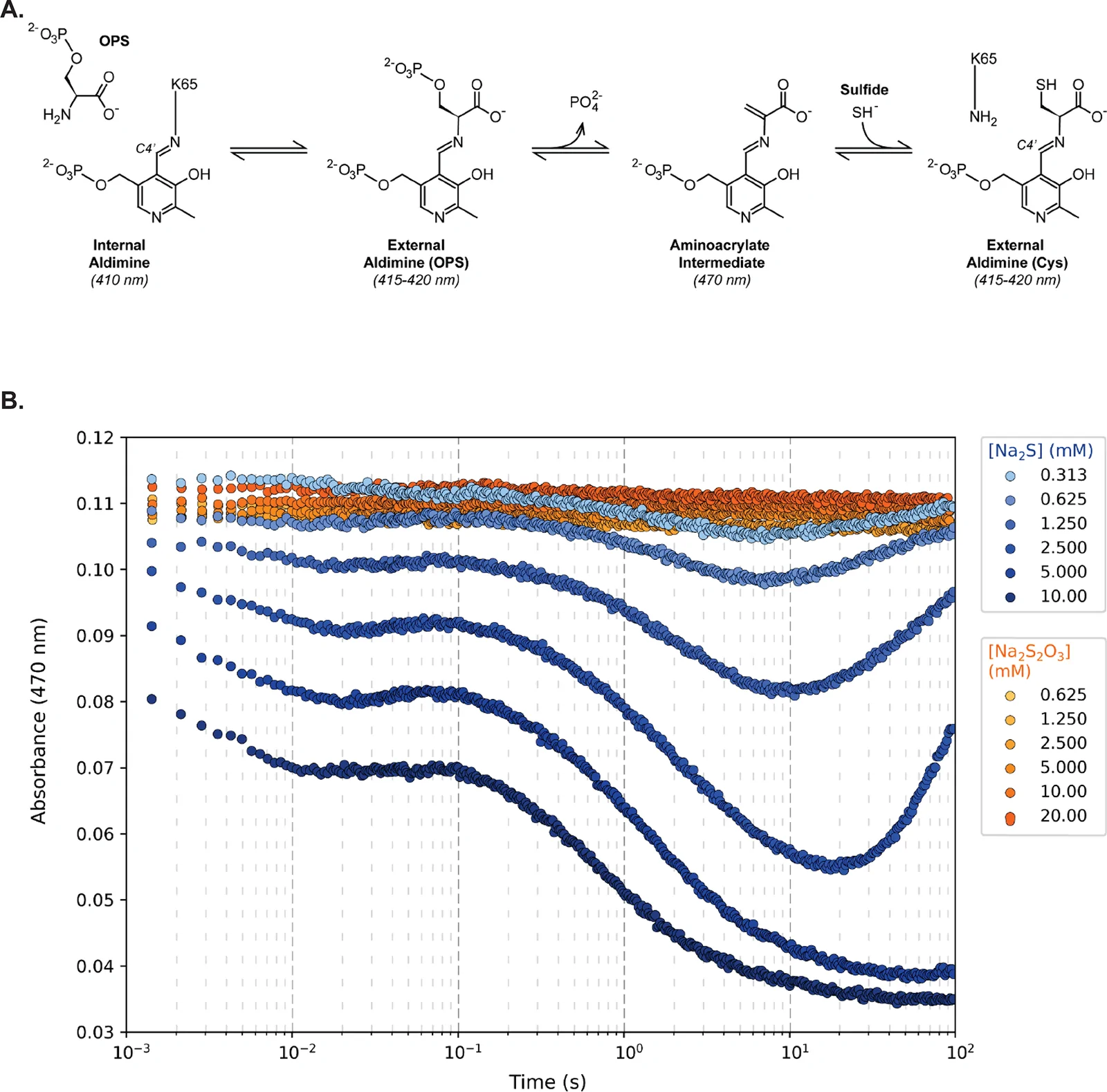
CysK2 uses sulfide as the sulfur donor substrate, not thiosulfate. (**A**) Schematic of proposed cysteine synthesis by CysK2. The reaction begins with OPS binding to the internal aldimine form of CysK2. The α-amino group of the substrate attacks the C4′ carbon of PLP to displace the enzyme (K65), forming the external aldimine. Deprotonation of the α-carbon of OPS triggers formation of the aminoacrylate intermediate and concomitant loss of the phosphate leaving group. Hydrogen sulfide is then able to add into the double bond to generate the external aldimine with cysteine. Attack of K65 on C4′ of the external aldimine releases cysteine and regenerates the resting state of the enzyme. (**B**) Comparison of the kinetics of sodium sulfide (Na_2_S) and sodium thiosulfate (Na_2_S_2_O_3_) reacting with the aminoacrylate intermediate of CysK2_S93_ monitored at 470 nm. CysK2_S93_ was mixed with 5 mm OPS and aged for 20 s before being mixed with Na_2_S (blue family of traces) or Na_2_S_2_O_3_ (orange family of traces).

Our kinetics studies of the aminoacrylate intermediate of CysK2_S93_ (i.e., the wild-type enzyme after reacting with OPS, as shown in Fig. 4A) indicate that sulfide reacts rapidly, while thiosulfate does not react with CysK2_S93_. In double-mixing, transient kinetics experiments, CysK2_S93_ was first mixed with OPS to form the aminoacrylate intermediate, which, in the second mixing step, was mixed with varied concentrations of either sodium sulfide (Na_2_S) or sodium thiosulfate (Na_2_S_2_O_3_; Fig. 4B, traces in blue [sulfide] and orange [thiosulfate], respectively). CysK2_S93_ did not react significantly with Na_2_S_2_O_3_. Even at mM concentrations of Na_2_S_2_O_3_, the absorbance at 470 nm due to the aminoacrylate intermediate did not begin to decrease until ~0.3 s after the second mixing step. Na_2_S, in contrast, reacted so quickly with the aminoacrylate intermediate that a significant portion of the reaction occurred within the ~0.001 s deadtime of the stopped-flow instrument (Fig. 4B, blue traces). The complicated, multiphasic behavior of the data was likely due to the multiple-turnover conditions used in these experiments. Our observation that sulfide, rather than thiosulfate, is the preferred sulfur-donor substrate of CysK2 differs from the observations of Steiner et al*.* (23). In experiments with the G93 variant, they found that thiosulfate reacted more readily than sulfide. More detailed kinetics studies of the G93 variant are required to answer this discrepancy.

**Fig 5:**
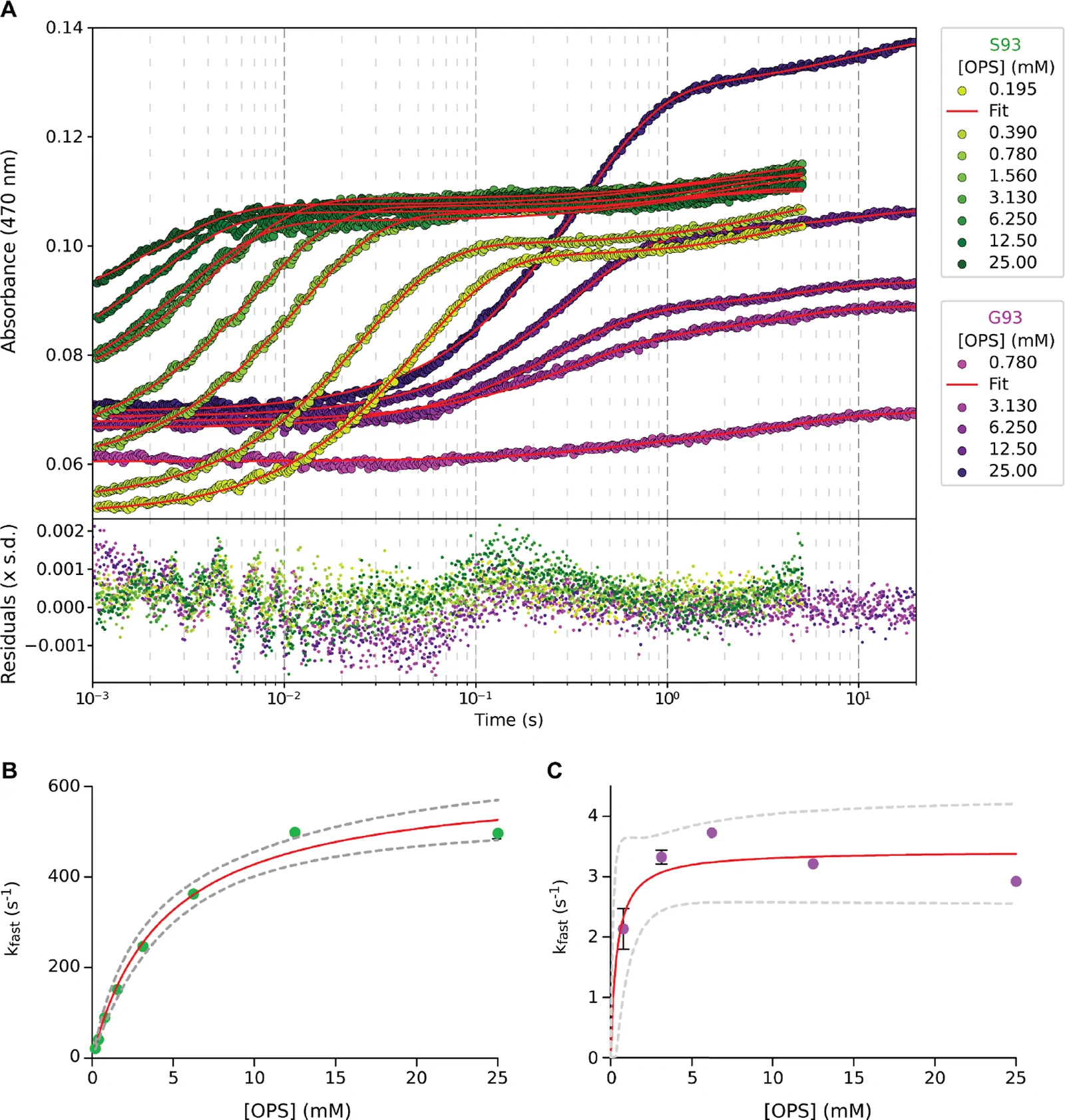
CysK2_S93_ is significantly more reactive toward OPS than the S93G variant. Kinetics of aminoacrylate formation from *O*-phospho-L-serine (OPS) catalyzed by CysK2_S93_ and CysK2_G93_. (**A**) Stopped-flow kinetics data showing the accumulation of the aminoacrylate intermediate monitored at 470 nm in the presence of CysK2_S93_ (green family of traces) and CysK2_G93_ (purple family of traces). Concentrations of OPS ranged from 0.195 to 25 mM. Fits of each kinetic trace to equation 1 are shown at red lines, and the residuals of those fits are shown in the subplot as multiples of the standard deviation. (**B**) Observed rate constants (*k*_fast_) of the fast phase of the CysK2_S93_ reaction plotted against the substrate concentration and fit to equation 2 to give *k*_max_ = 620 ± 32 s^−1^ and *K*_1/2_ = 4.49 ± 0.66 mM. The gray dotted lines represent the 95% CI bounds. (**C**) Dependence of *k*_fast_ on the concentration of OPS for the CysK2_G93_ reaction. Fitting the data to equation 2 gives *k*_max_ = 3.4 ± 0.3 s^−1^ and *K*_1/2_ = 0.37 ± 0.25 mM.

CysK2_S93_ reacted with OPS approximately 180-fold faster than CysK2_G93_ (Fig. 5). The reactions of each CysK2 variant were investigated with single-mixing, transient kinetics experiments where the formation of the aminoacrylate intermediate was monitored by absorbance at 470 nm (Fig. 5A). The increase in absorbance at 470 nm was biphasic in all cases, with a rapid first phase, followed by a slow second phase. With CysK2_S93_, even at the lowest concentration of OPS tested (0.195 mM), the fast phase began at the earliest time points (~1.4 ms). As the concentration of OPS increased, the proportion of the fast phase absorbance change occurring during the deadtime of the instrument also increased (Fig. 5A, green traces). At the highest concentration of OPS tested (25 mM), approximately half of the fast absorbance change occurred during the deadtime. Note in Fig. 5A that the green traces begin higher on the *Y*-axis as the concentration of OPS increases. CysK2_G93_, in contrast, does not begin accumulating aminoacrylate until ~10 ms after mixing with 25 mM OPS (Fig. 5A, purple traces). Note also that, whereas the CysK2_S93_ data all finish with roughly similar maximum absorbance changes, the total absorbance change with CysK2_G93_ increases with the concentration of OPS. All traces were fit to the linear combination of exponentials (equation 1) described in Materials and Methods. The *k*_fast_ values were extracted and replotted against the concentration of OPS for both CysK2_S93_ and CysK2_G93_ (Fig. 5B and C, respectively). Both *k*_fast_ plots were fitted to the hyperbolic equation 2 to yield an estimate of *k*_max_. The *k*_max_ value for CysK2_S93_ was estimated at 620 ± 32 s^−1^, 180-fold higher than the value of 3.4 ± 0.3 s^−1^ estimated for CysK2_G93_.

**Fig 6:**
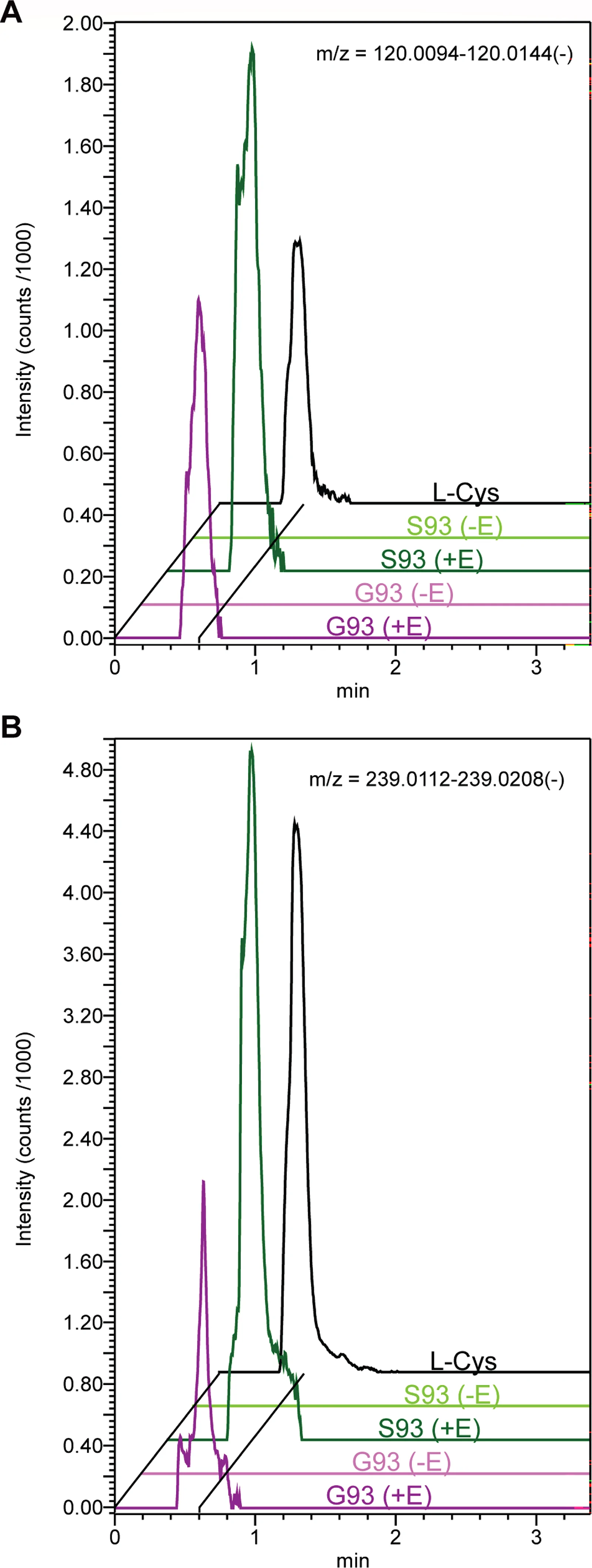
Both CysK2 alleles produce L-Cys from OPS and sodium sulfide. Targeted quadrupole time-of-flight (QTOF) LC-MS analysis of CysK2_S93_ and CysK2_G93_ reaction mixtures showing that the enzyme produces L-Cys from OPS and Na_2_S. (**A**) Negative-mode ([*M-H*]^―^) extracted-ion chromatograms (EIC) for the deprotonated molecular ion of L-Cys at *m*/*z* = 120.01193 using a ±20 ppm window. The black chromatogram (top) belongs to the 500 μM L-Cys standard. The light green and dark green chromatograms are from reactions containing 500 μM OPS, 5 mM Na_2_S, and boiled (−E) or active (+E) CysK2_S93_, respectively. The magenta and purple lines are chromatograms from reactions containing the same substrate concentrations and boiled (−E) or active (+E) CysK2_G93_, respectively. The chromatograms are vertically and horizontally offset to increase clarity. (**B**) Negative-mode EICs from the same samples for the deprotonated molecular ion of L-cystine at *m*/*z* = 239.01603 using a ±20 ppm window. Note that L-cystine is detected due to partial oxidation of L-Cys during the LC-MS analysis.

High-resolution mass spectrometry analysis indicates that the reaction between OPS and Na_2_S catalyzed by both CysK2 variants produces L-Cys (Fig. 6). Reaction mixtures were analyzed for the presence of L-Cys (Fig. 6A) or L-cystine (Fig. 6B), which forms due to oxidation of L-Cys during the liquid chromatography-mass spectrometry (LC-MS) analysis. All reactions containing active CysK2_S93_ or CysK2_G93_ were found to contain both L-Cys and L-cystine. The mass specificity and retention time match with the authentic L-Cys standard establish the identity of the CysK2 product as L-Cys. Reactions containing heat-inactivated CysK2 ("-E") had no detectable L-Cys or L-cystine.

## DISCUSSION

In this study, we identified a serine to glycine substitution at position 93 of the cysteine synthase CysK2 in H37Rv that contributes to its increased copper sensitivity compared to other commonly studied *Mtb* strains. CysK2_G93_ in H37Rv is less enzymatically active when compared to the ancestral, wild-type CysK2_S93_ and consequently provided less protection against copper toxicity *in vitro*. While *cysK2* has been reported to be a part of the RicR copper-responsive regulon (20), this study is the first to demonstrate its role in copper resistance.

Previous work (23) using the mutant CysK2_G93_ concluded that it produced S-sulfocysteine from OPS and thiosulfate. In contrast, we found that both CysK2_S93_ and CysK2_G93_ catalyzed the formation of cysteine from OPS and sulfide. Furthermore, we found no evidence of a reaction between the CysK2_S93_ aminoacrylate and thiosulfate. Our preliminary kinetics analysis indicated that CysK2_G93_ is significantly less reactive with OPS than the ancestral CysK2_S93_. Our mass spectrometric analysis confirms that both CysK2 variants can produce L-Cys from OPS and sulfide in end-point assays. The presence of L-cystine is due to oxidation of L-Cys occurring during the LC-MS analysis, which is supported by the observation that L-cystine was also detected in the cysteine standard. Our conclusion that CysK2_G93_ is enzymatically hypoactive compared to the ancestral CysK2_S93_ is consistent with both our *in vitro* observations with copper and *in vivo* results of Le Mouëllic et al. (26).

We began this study based on previous observations that *Mtb* strain H37Rv is more sensitive to copper restriction than strains CDC1551 and Erdman. Within this study, we found that CDC1551 is even more resistant to copper than *Mtb* strain Erdman. Qualitatively, deletion of *cysK2* also reduced copper resistance in CDC1551 more, and complementation with *cysK2_G93_* provided less resistance than was seen for Erdman. It is possible that there are other mechanisms relating to cysteine or sulfur regulation pathways that contribute to these observed differences between the strains. Copper-binding proteins often use cysteine residues to coordinate copper. Relatedly, iron-sulfur clusters that require cysteine are targets of copper toxicity (14, 15); thus, an increased production of cysteine might provide a protective buffer in the presence of excess copper in *Mtb*. It is also possible that there may be mechanisms affecting copper susceptibility that are unlinked to RicR or CsoR regulons or cysteine metabolism and remains to be explored.

Despite significant differences in *in vitro* assays between strains with CysK2_G93_ and CysK2_S93_ or strains with CysK2_S93_ and those without, we were unable to find any significant differences *in vivo* between any of the strains on either H37Rv or CDC1551 background. Previous studies on copper response genes (7, 8) have observed similar trends, where significant *in vitro* attenuation by a single gene deletion does not translate into differences in mice. Shi et al. (7) found significant attenuation in mice only by using a strain producing a RicR allele that does not respond to copper, in essence repressing the entire RicR regulon, even in the presence of copper. Wolschendorf et al. (8) observed significant differences with an *mctB* mutant compared to its parental strain in a guinea pig model, but only a subtle phenotype in mice, suggesting that the mouse model may not be an optimal model for studying *in vivo* copper-related phenotypes in *Mtb*.

Although we did not observe any differences in bacterial burden with any of our strains, Le Mouëllic et al. observed a subtle but significant attenuation with an H37RvΔ*cysK2* strain in mice (26). It is possible that attenuated virulence from the loss of CysK2_G93_ is more apparent using a low-dose infection model as described by Le Mouëllic et al. (26), whereas our "medium" infection dose of 200–400 CFU/mouse was unable to reveal these differences. The subtle attenuation, along with our enzymology data, suggests that CysK2_G93_ retains some activity. In another study, Khan et al. (27) hypothesized multiple cysteine synthetic pathways were important for virulence and reported a near 100-fold attenuation of an H37RvΔ*cysK2* strain compared to the parental and complemented strains in mice using an infection dose of around 200 CFU/mouse, similar to what we used in our work. While we cannot explain this conspicuous discrepancy in animal data, it is possible experimental differences resulted in a different outcome. Notably, Khan et al. (27) did not consider RicR-dependent regulation of *cysK2* or copper resistance as a mechanism of CysK2 activity.

In an age of increasing antibiotic resistance, there is renewed interest in novel uses of copper as an antimicrobial agent (reviewed in 28). Copper synergizes with disulfiram and capreomycin against *Mtb* (29, 30). Disulfiram increases cytosolic copper accumulation, possibly through a shuttling mechanism (29). Our results suggest that candidate inhibitors of cysteine synthases (31, 32) could potentially synergize with antimicrobials that induce copper stress in *Mtb*. Interestingly, *Mtb* encodes numerous toxin-antitoxin systems, including a toxin, VapC4, that targets tRNA^Cys32^. Depletion of this cysteine-charged tRNA results in the upregulation of genes in the CsoR and RicR regulons, including *cysK2* (33), suggesting that copper responses and cysteine availability are coregulated through mechanisms that require further elucidation. Finally, the identification of virulence-associated, copper acquisition pathways (9) during copper insufficiency further solidifies a crucial role of copper in *Mtb* pathogenesis.

## MATERIALS AND METHODS

### Bacterial strains, growth conditions, plasmids, and primers

The bacterial strains, plasmids, and primers used in this work are listed in Table 1. *Mtb* strains were grown in 7H9 liquid media (Difco) supplemented with 0.2% glycerol, 0.05% Tween 80, 0.5% bovine serum albumin (BSA), 0.2% dextrose, and 0.085% sodium chloride (ADN) ("7H9c"). Cultures were grown at 37°C without agitation in vented flasks (Corning). For *M. tuberculosis* growth on solid media, Middlebrook 7H11 agar (Difco and Remel) was supplemented with Middlebrook OADC (oleic acid, albumin, dextrose, and catalase; BBL). *Mtb* strains were grown in 50 µg/mL kanamycin, 25 µg/mL streptomycin, or 50 µg/mL hygromycin when necessary.

For CuSO_4_ solutions, stock solutions were made by dissolving the appropriate amount of CuSO_4_ powder (Fisher Scientific) in water and sterilizing with a 0.22 µm filter.

All plasmids were sequenced by Azenta, Inc. to confirm the veracity of PCR-generated clones. All enzymes were purchased from New England Biolabs (NEB), Inc. We used Phusion Polymerase (NEB) and Q5 High-Fidelity DNA Polymerase (NEB) for PCR products used for cloning and Qiagen Taq Polymerase for screening allelic exchange clones.

The *cysK2*::MycoMarT7 mutant was isolated from a library of ordered transposon insertion mutants as previously described (34, 35). To make ∆*cysK2::hyg* strains, we deleted and replaced *cysK2* using a previously described method (20). Briefly, pYUB854 (25) was used to clone sequences encompassing ~800 bp upstream (5′) and ~800 downstream (3′) of the *cysK2* gene. The 5′ and 3′ sequences, including the start and stop codons, respectively, were cloned to flank the hygromycin resistance cassette in pYUB854. The plasmid was digested with PacI, and approximately 1 µg of linearized, gel-purified DNA was used for electroporation into *Mtb. Mtb* strains were grown to an OD_580_ of ~0.4 to 1, washed, and resuspended in 10% glycerol to make electrocompetent cells as described in detail in reference 36. Bacteria were inoculated onto 7H11 agar with 50 µg/mL hygromycin as needed; a no-DNA control electroporation was done to control for spontaneously antibiotic-resistant mutants. Two weeks after plating, colonies were picked and inoculated into 200 µL 7H9c with antibiotics, cultured for 1–2 weeks, and then inoculated into 5-mL cultures for further analysis. Mutants were confirmed by PCR and sequence analysis.

### Bioinformatic search for mutations in H37Rv, CDC1551, and Erdman

We compared amino acid and genomic sequences of ORFs previously identified as copper-responsive or involved in copper resistance (19, 20). Protein sequences were derived from National Center for Biotechnology Information (NCBI) protein database using the reference H37Rv proteome. Wherever possible, we used NCBI Identical Protein Groups database to search for whether proteins were identical to that of H37Rv.

Corresponding CDS region in nucleotide of each protein in the NCBI database was used to retrieve relevant genomic sequences for each protein. We included 500 bp up- and downstream of each coding region. The genome assemblies used were: CDC1551 NC_002755.2, Erdman NC_020559.1, and H37Rv NC_000962.3. The comparisons included: Rip1 (Rv2869c, NP_217385.1), PdtaS (Rv3220c, NP_217736.1), PdtaR (Rv1626, NP_216142.1), CsoR (Rv0967, NP_215482.1), Rv0968 (NP_215483.1), CtpV (Rv0969, NP_215483.1), Rv0970 (NP_215485.1), RicR (Rv0190, NP_214704.1), MymT (Rv0186A, YP_004837046.2), MmcO (Rv0846c, NP_215361.1), LpqS (Rv0847, NP_215362.1), CysK2 (Rv0848, CysK2_G93_ YP_177762.1, CysK2_S93_ WP_003404395.1), and Rv2963 (NP_217479.1). Proteins corresponding to the RicR-regulated genes SocAB were not annotated in the H37Rv proteome, so we compared nucleotide sequences from the known upstream gene PPE23 (Rv1706c) to the downstream gene Rv1706A.

Of the proteins compared, CysK2 was the only one with a non-synonymous amino acid substitution that distinguished the copper-sensitive H37Rv from copper-resistant CDC1551 and Erdman.

### Mouse infections

Mouse infections were performed essentially as described previously (34). *Mtb* strains were grown in 7H9c medium to an OD_580_ of ~0.4, and clumps were removed from the cultures by centrifugation at 800 × *g* for 8 min. Six- to eight-week-old mice (C57BL6/J, Jackson Laboratories) were infected with a Glas-Col Inhalation Exposure System to inoculate ~200 to 400 CFU/mouse. Mice were humanely euthanized, and organs were harvested at days 1, 21, and 56 (*n* = 6 for day 1, 8 for days 21 and 56 over two independent experiments) following infection. Lungs were harvested at all time points, and spleens were harvested at all time points except day 1. Samples were homogenized and inoculated onto 7H11 agar. CFU were enumerated after 2 to 3 weeks.

### Copper sensitivity assays

The copper sensitivity assay was performed as described previously (21, 37). *Mtb* strains were grown in 7H9c medium to an OD_580_ of ~0.5 to 1. Cells were harvested by centrifugation, washed once with PBS-Tween (0.05%), and then spun slowly at 800 × *g* for 8 min. Declumped cell suspensions were diluted to an OD_580_ of 0.1 with PBS-Tween. Three-microliter samples of serial dilutions were spotted onto 7H11-OADC agar plates containing either no additional CuSO_4_ or different concentrations thereof. Plates were incubated at 37°C for ~2 to 3 weeks.

### Purification of CysK2_S93_ and CysK2_G93_

The *cysK2*_S93_ and *cysK2*_G93_ genes were amplified from the respective plasmids pQE30-CysK2_S93_ or pQE30-CysK2_G93_ using the same primer pair (CysK2_pSUf and CysK2_pSUr, Table 1) and subcloned into the pE-SUMOpro(kan) vector (LifeSensors, Inc.) in frame with the His_6_-SUMO tag. The sequences of positive clones were confirmed by sequencing the *cysK2*_S93_ or *cysK2*_G93_ genes. The resulting pSUMO-*cysK2*_S93_ and pSUMO-*cysK2*_G93_ plasmids were transformed into *E. coli* BL21 Star (DE3) cells (Thermo Fisher/Invitrogen) for expression.

Colonies of BL21 Star (DE3):pSUMO-*cysK2* were used to inoculate 20 mL of Luria-Bertani (LB) broth (Miller) (10 g/L casein peptone, 5 g/L yeast extract, 10 g/L NaCl) supplemented with 50 μg/mL kanamycin. These starter cultures grew overnight at 37°C with 230 rpm agitation. The following day, the starter cultures were used to inoculate 1 L portions of LB + Kan. The large-scale cultures were grown at 37°C and 230 rpm until OD_600_ of 0.6–0.8 and then grown for 30 min with shaking at 22°C. Expression of the His_6_-SUMO-CysK2 fusion proteins was induced by the addition of 0.4 mM IPTG. The cells were allowed to express protein overnight at 22°C at 230 rpm.

Bacteria were then harvested by centrifugation at 5,300 × *g* for 20 min and resuspended in buffer A (25 mM Tris, pH 8.0, 300 mM NaCl, 10 mM imidazole) supplemented with 200 μM pyridoxal 5′-phosphate (PLP) and 10 μg/mL DNase I (Worthington Biochemical). Once fully resuspended, the cells were transferred to a steel beaker, suspended in a stirred ice bath, and lysed using a Branson 450 sonic dismembrator set at 60% amplitude for 20 cycles of sonication (30 s) and rest (30 s). The resulting lysate was clarified by centrifugation at 39,000 × *g* for 45 min. The lysate was passed through a 0.45 μm glass-prefiltered syringe filter before being loaded onto a 5 mL HisTrap cartridge (Cytiva) installed on an Äkta GO FPLC that had been pre-equilibrated with buffer A (note that PLP was not included in the running buffer). HisTrap runs were limited to 30 mL of lysate to avoid overloading the resin. The purification program entailed a three-step gradient of a five column-volume (CV) wash with 5% buffer B (25 mM Tris, pH 8.0, 300 mM NaCl, 250 mM imidazole), followed by a five CV wash with 15% buffer B, and, finally, a five CV elution step with 100% buffer B. The elution fractions containing the His_6_-SUMO-CysK2 fusion protein were bright yellow owing to the PLP and generally about 90% pure based on Coomassie-stained SDS-PAGE gels (12% polyacrylamide).

The elution fractions from all HisTrap chromatography runs were pooled in an appropriate length of SnakeSkin dialysis tubing (Pierce), and 2 mL of 150 μM SUMO protease was added. The fusion protein-protease mixture was dialyzed against 4 L of SUMO protease buffer (50 mM Tris, pH 8.0, 150 mM NaCl, 0.5 mM DTT, 200 μM PLP) for 18 h at 4°C. This step removes the His_6_-SUMO tag from CysK2 and reduces the imidazole concentration to <1 mM. Inclusion of PLP in the dialysis buffer is required to prevent precipitation of the CysK2. The dialysate was passed through the same HisTrap column used for the initial purification step. Owing to the removal of the His_6_-SUMO tag, the CysK2 was collected in the flow-through of the column during sample loading. The protein was judged to be approximately 95% pure at this stage. Fully purified CysK2_S93_ or CysK2_G93_ was dialyzed against 4 L of storage buffer (10 mM NaPO_4_, pH 6.3, 20 μM PLP) for 24 h at 4°C. The finished enzyme preparations were concentrated to approximately 25 μM and stored in 2 mL aliquots at −80°C.

### Pre-steady state kinetics analysis

All transient kinetics experiments were performed on a SF-61DX2 stop-flow spectrophotometer equipped with either a photomultiplier tube (PMT) detector or a CMOS array detector. All data reported here are the result of averaging at least three individual traces.

The kinetics of the sulfur donors, sodium sulfide (Na_2_S) or sodium thiosulfate (Na_2_S_2_O_3_), reacting with the CysK2_S93_ aminoacrylate intermediate was studied in double-mixing mode using the FX spectrometer CMOS array detector at 25°C. Fresh 100 mM stocks of *O*-phospho-L-serine, Na_2_S, and Na_2_S_2_O_3_ were prepared in ultrapure water. The OPS stock was adjusted to pH 8.0 with 10 M NaOH. All three substrate stocks were then diluted to 20 mM with 200 mM Tris at pH 8.0. The two sulfur donor substrates were each serially twofold diluted to give sets of solutions at 40, 20, 10, 5, 2.5, and 1.25 mM (Na_2_S_2_O_3_) or at 20, 10, 5, 2.5, 1.25, and 0.625 mM (Na_2_S). CysK2_S93_ (80 μM) in 10 mM sodium phosphate (NaPi), pH 6.3, and 20 μM PLP was first mixed with 20 mM OPS in 200 mM Tris at pH 8.0 and allowed to react for 20 s. After this age time, the reaction was mixed with an equal volume of Na_2_S or Na_2_S_2_O_3_ solution. The final concentrations after the second mixing step were 20 μM CysK2_S93_, 5 mM OPS, and 0.313 to 10 mM Na_2_S or 0.625 to 20 mM Na_2_S_2_O_3_. Spectral changes were monitored from 240 to 700 nm from 0.001 to 100 s.

The reactions of both CysK2 variants with OPS were investigated in single-mixing mode using the PMT detector to better sample the earliest times after mixing. A fresh OPS stock was prepared as above and twofold serially diluted from 50 to 0.39 mM. CysK2_S93_ (25 μM in NaPi buffer with 20 μM PLP) was mixed with each of the OPS solutions, in turn, from the lowest to the highest concentration (0.195–25 mM, after mixing). Accumulation of the aminoacrylate intermediate was monitored at 470 nm from 0.001 to 5 s. The experiment was repeated with CysK2_G93_ in the same manner, save that the reaction was monitored from 0.001 to 20 s. In both cases, the 470 nm traces were fit in GraphPad Prism 15 to linear combinations of two exponentials to capture the fast and slow phases observed in the data. In this equation (equation 1), *A*_470nm_ is the observed absorbance value; Δ*A*_fast_ is the absorbance change associated with the fast phase; *k*_fast_ is the rate constant for the fast phase; Δ*A*_slow_ is the absorbance change for the slow phase; *k*_slow_ is the associated rate constant; and *C* is the endpoint of all absorbance changes. These fits are shown throughout as solid red lines. The values of *k*_fast_ and the associated parameter error estimates from fitting were plotted against OPS concentration and fit to equation 2 to extract estimates of *k*_max_ and *K*_1/2_ for each enzyme.


(1) $$A_{470}=\Delta A_{fast}\left(1-e^{-k_{fast}t}\right)+\Delta A_{slow}\left(1-e^{-k_{slow}t}\right)+C$$

(2) $$k_{fast}=\frac{k_{\max}[OPS]}{K_{1/2}+[OPS]}$$

### Mass spectrometry analysis of CysK2_S93_ and CysK2_G93_ reaction mixtures

Fresh stocks of OPS and Na_2_S were prepared at 100 mM in ultrapure water immediately before use. Reactions (1 mL) were assembled containing 500 μM OPS, 5 mM Na_2_S, 10 μM enzyme (CysK2_S93_ or CysK2_G93_), and 10 mM Tris at pH 8.0. Heat-inactivated controls were generated by heating aliquots of each enzyme for 5 min at 80°C, followed by centrifugation for 5 min at ~16,000 × *g* to remove precipitated protein. The reactions stood at RT for 2 h, after which they were heated to 80°C to quench activity and centrifuged to remove particulates. The clarified supernatants were filtered through 10 kDa MWCO centrifugal concentrators. Each filtrate was diluted 1:2 (vol/vol) with 12 mM HCl prior to LC-MS analysis. Control samples included 500 μM L-Cys, 500 μM OPS, and 10 mM Tris, pH 8.0 (buffer only).

The chromatographic separations were performed using a Kinetex EVO-C18 column (Phenomenex; 1.7 μm, 100 Å, 50 × 2.1 mm) installed on a Shimadzu LCMS-9030 QTOF instrument. Buffer A consisted of 0.1% trifluoroacetic acid in water; buffer B was neat acetonitrile. The 8-min program started at 0% buffer B for 2 min, followed by a linear gradient from 0 to 100% buffer B over 5 min, a decrease back to 0% buffer B over 0.1 min, and a 1 min re-equilibration at 0% buffer B. The flow rate was 0.4 mL/min.

Samples were ionized using electrospray ionization (ESI) and analyzed in negative-ion mode. The instrument settings used were as follows: interface voltage, −3.0 kV; interface temperature, 300°C; desolvation line temperature, 250°C; heat block temperature, 400°C; drying gas flow, 10 L/min; heating gas flow, 10 L/min; nebulizing gas, 1.5 L/min; and detector voltage, 1.74 kV. Targeted extracted ion chromatograms (EICs) were generated with a ±20 ppm mass window for the deprotonated molecular ions of L-Cys ([*M-H*]^–^ = *m*/*z* 120.001193) and L-cystine ([*M-H*]^–^ = *m*/*z* 239.01603). Authentic L-Cys, OPS, and Tris standards (500 µM each) were run under identical LC-MS conditions to confirm retention times and accurate masses.
